# X-linked FRMD7 gene mutation in idiopathic congenital nystagmus and its role in eye movement: A case report and literature review

**DOI:** 10.3389/fopht.2022.1080869

**Published:** 2023-03-06

**Authors:** Fanfei Liu, Minjin Wang, Meng Liao, Longqian Liu, Xiaoshuang Jiang

**Affiliations:** ^1^ Department of Ophthalmology, West China Hospital of Sichuan University, Chengdu, Sichuan, China; ^2^ Department of Laboratory Medicine, West China Hospital of Sichuan University, Chengdu, Sichuan, China

**Keywords:** idiopathic congenital nystagmus, FRMD7, X-linked, gene mutation, eye movement

## Abstract

**Background:**

Idiopathic congenital nystagmus (ICN) is an inherited disorder characterized by uncontrollable binocular conjugating oscillation. X-linked idiopathic congenital nystagmus is one of the most prevalent types of ICN. Elucidation of the genetic mechanisms involved in ICN will enhance our understanding of its molecular etiology.

**Case presentation:**

We report a girl with uncontrollable binocular oscillation and anomalous head posture, then presented a novel heterozygous missense variant (c.686G>T) within the mutation-rich region of the FERM domain containing 7 (FRMD7) gene in her family member. The girl received occlusion therapy and surgical operation which balanced her binocular vision and corrected the anomalous head posture.

**Conclusions:**

This is the first report on a mutation (c.686G>T) caused the substitution of Arg (R) with Leu (L) at position 229 (p.R229L) of the FRMD7 protein in a patient with ICN.

## Background

Idiopathic congenital nystagmus (ICN) is a disorder characterized by uncontrollable binocular conjugating oscillation that occurs in the horizontal or vertical plane of either a jerking or pendular waveform. These pathophysiological events progress commonly at birth or shortly afterward (1). Genetically, ICN is a heterogeneous disease that could be divided into autosomal dominant (2), recessive (3, 4), and X-linked dominant (3), with X-linked type being the most prevalent (5). This disorder may be caused by deregulation of signaling pathways or mutation of a single gene (6). Two major genes, FERM domain containing 7 (FRMD7) and G-protein coupled receptor 143 (GPR143), have been implicated in the pathogenesis of ICN (7, 8). Studies have demonstrated that FRMD7 is expressed in the retina, cerebellum, lateral ventricles, and neurite during development (6). Several distinct X-linked loci have been reported to be associated with congenital nystagmus: Xp11.4-p11.3 (4), Xq26-Xq27 (3), Xp22.3-p22.2 (9), and Xq24-q26.3 (10). Approximately 20–97% of patients with X-linked ICN harbor FRMD7 mutations at Xq26.2 (11).

ICN is highly associated with the occurrence of low visual acuity, which is attributed to formation of an unstable image away from the fovea (1). Hypothetically, ICN could be a developmental response to incomplete high-contrast foveal vision (12). Most patients show a fixed angle where they achieve the best visual acuity and turn their heads in the opposite direction of the null zone. Currently, the pathogenesis of ICN has not been fully established. Although there is no etiological treatment for ICN, surgery is often performed to improve patient symptoms. We investigated a five-generation Chinese family with a history of ICN. Surgery was performed on a child diagnosed with ICN to improve anomalous head posture. A summary of recent studies exploring the association of FRMD7 gene mutation with ICN, as well as the role of FRMD7 plays in the regulation of eye movement is presented in this report.

## Case presentation

**Figure 1:**
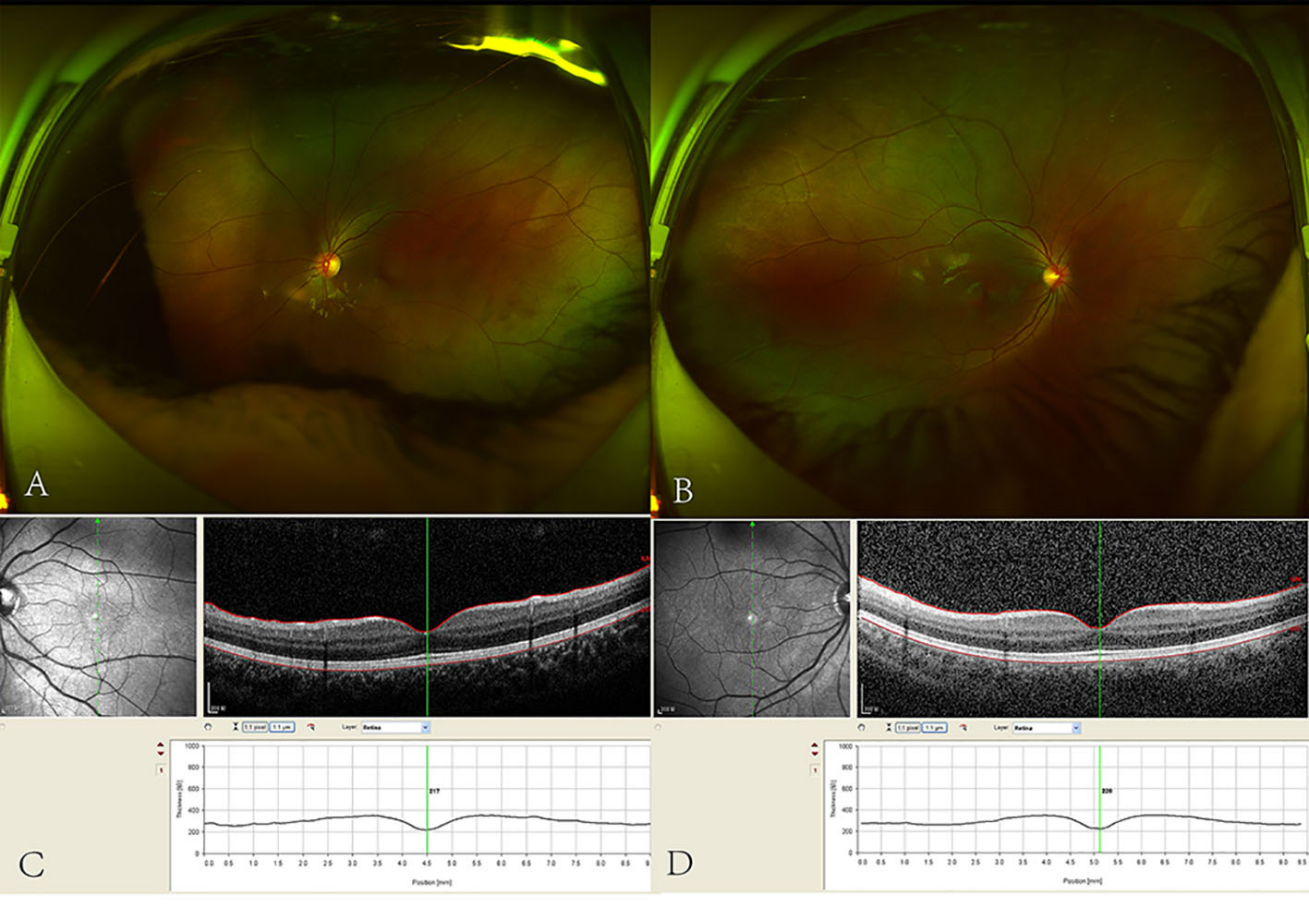
Fundus photography and OCT of the four-year-old Chinese girl. **(A)** Fundus photography of left eye. **(B)** Fundus photography of right eye. **(C)** OCT of the macular fovea and thickness of retinal layer on left eye. **(D)** OCT of the macular fovea and thickness of retinal layer on right eye.

This case report was approved by the ethics committee of the West China Hospital of Sichuan University and written informed consents were obtained from the patients. A four-year-old Chinese girl who presented with uncontrollable binocular oscillation and anomalous head posture was referred to the West China Hospital of Sichuan University. She had no significant past ophthalmic or medical history. On admission, her corrected visual acuity (BCVA) was 20/66 with +1.00Dc×90° in the right eye and 20/40 with +0.50 Dc×90° in the left eye. Her nystagmus was pendular and horizontal. Her face turned right to get optimal VA. No remarkable abnormalities were observed in the anterior segments of both eyes. Findings of the fundus exam and optical coherence tomography (OCT) revealed that thickness measurements of the retinal layer and macular fovea were normal (**Figure 1**). Moreover, titmus test showed no near stereopsis. No other general physical and neurological symptoms were observed.

Evidence from previous studies demonstrate that refractive correction could improve visual acuity and reduce nystagmus in ICN (13).We gave the four-year-old Chinese girl a prescription for glasses and followed up. At one-year follow-up, her BCVA was 20/50 in the right eye and 20/30 in the left eye. Moreover, occlusion was observed in the left eye. At two-year follow-up, monocular BCVA was 20/30 in each eye. Binocular BCVA was 20/20 when she looked left, 20/30 when she looked forward, and 20/66 when she looked right.

Subsequently, surgery was performed on both eyes targeting the internal and external rectus muscles at the age of six. Specifically, the lateral rectus was recessed 10.0 mm from the insertion in the left eye, and the medial rectus was recessed 10.5 mm from the limbus in the right eye. One month after surgery, her compensatory head posture was improved, and the null zone was in the straight-ahead gaze; her binocular BVCA improved to 20/25 when she looked forward; the near and far binocular vision were restored. The titmus test revealed the occurrence of near gross stereopsis. Binocular vision examined using the synoptophore (Inami, Japan) showed a simultaneous perception at 0°, fusional amplitude ranging from -6° to +10°, and far stereopsis in the range of -5° to +10°. Her head posture was corrected to normal. At the age of nine years, her binocular BCVA was 20/25 when she looked forward and left. Surgery could shift the null zone of nystagmus into primary position (14), and recession or tenotomy of the four horizontal muscles could significantly improve visual function and eye movements in ICN patients (15). This study provide evidence that extraocular surgery intervention on two horizontal muscles could independently improve neurological and visual quality to achieve eye repositioning. The intervention on two horizontal muscles which also have been peformed by Dr.Muralidhar in patients with infantile nystagmus syndrome and face turn (16).

**Figure 2:**
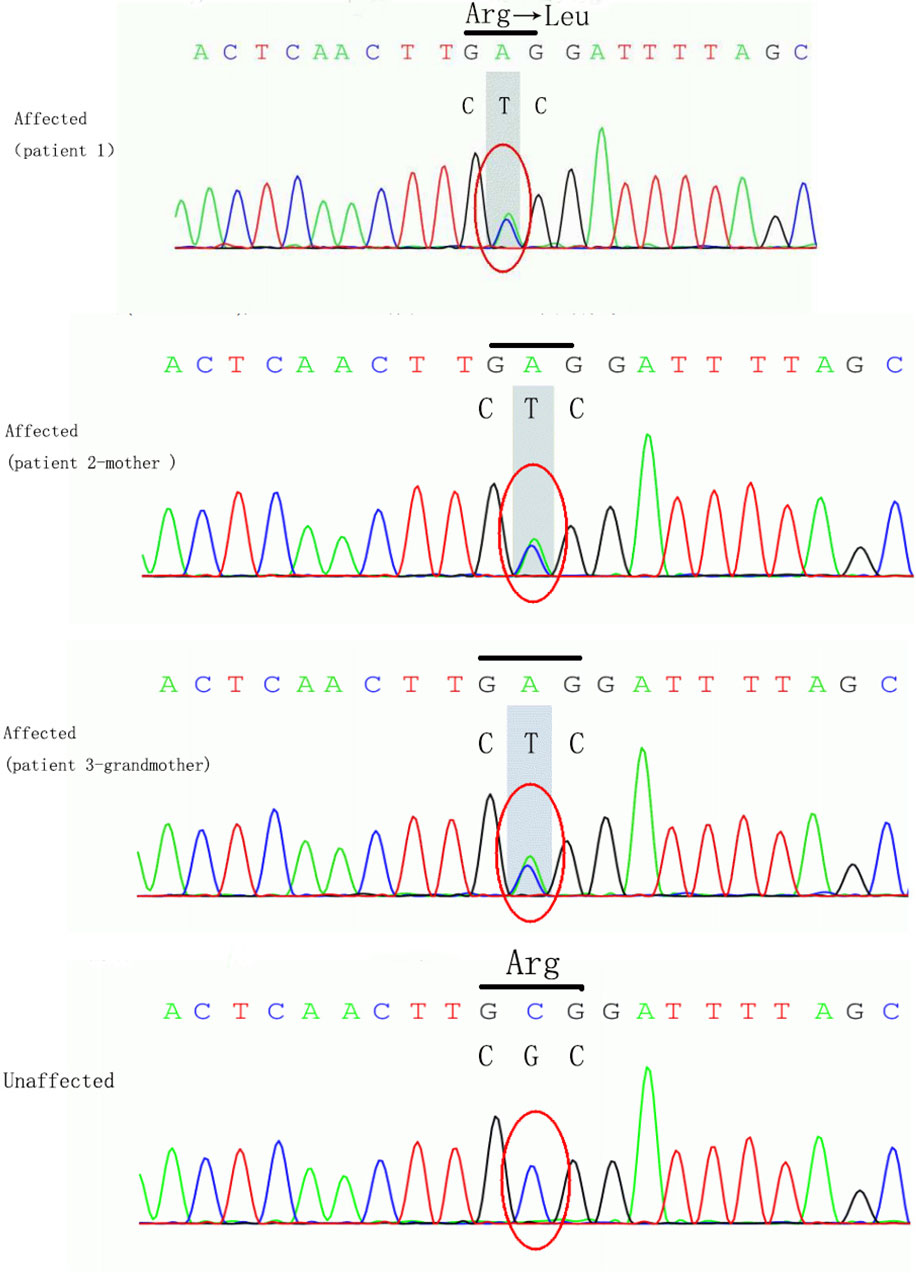
The loci of mutation in FRMD7. The mutation c.686G>T caused a substitution of Arg (R) to Leu (L) at position 229 (p.R229L) of the FRMD7 protein in the girl, her mother and grand-mother.

**Figure 3:**
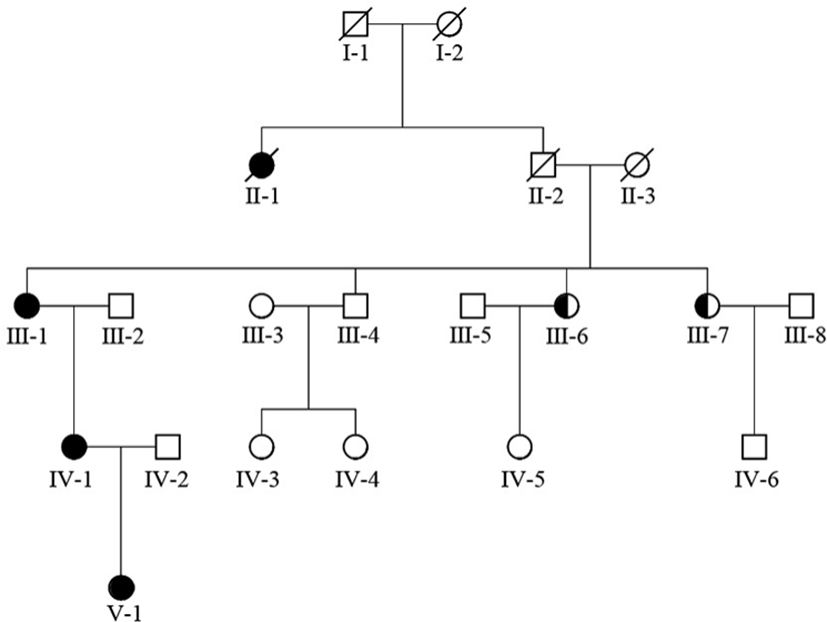
FRMD7 mutations in this family. The family (20 members) included four affected females and two carriers.

The girl in the present case came from a five-generation Chinese family, in which the mother and maternal grandmother had nystagmus, but the other family members did not develop nystagmus. Binocular BCVAs of the patient’s mother and maternal grandmother were 20/50, and both exhibited anomalous head postures. However, they did not undergo surgical treatment. None of the family members showed remarkable structural abnormalities in the eyes. To further investigate the underlying genetic cause of nystagmus, we scheduled a genomic DNA test for member of the family. Consequently, peripheral venous blood samples were collected from each family member after obtaining written informed consent. Genomic DNA was extracted from the blood samples, whole exome was isolated using the Agilent SureSelect Human All Exon Kit and then sequencing on the Agilent Hiseq sequencer. A novel missense heterozygous mutation, c.686G>T, was identified at codon 686 in exon 8 of the FRMD7 gene (**Figure 2**). The c.686G>T mutation caused a substitution of Arg (R) with Leu (L) at position 229 (p.R229L) of the FRMD7 protein. A gene test revealed that among the 20 family members, four females were affected and two members were carriers (**Figure 3**). The mutation was detected in the patient’s mother, maternal grandmother, and two sisters of the maternal grandmother (**Figure 2**).

## Discussion and conclusions

Congenital nystagmus is a clinically and genetically heterogeneous disorder that affects vision (1). This disorder is characterized by idiopathic and nystagmus-related disorders. It has been postulated that ICN might be caused by a developmental defect in the brain’s ocular motor regions that control fixation (1).

Here, we identified a novel heterozygous missense mutation of the FRMD7 gene in a Chinese family with ICN. A four-year-old Chinese girl who had ICN underwent surgical treatment to reposition of the minimal intensity zone to the primary position of the eye. The surgery balanced binocular vision and corrected the anomalous head posture at 3-year follow-up. The identified mutation caused substitution of CGC (that codes for Arg) with CTC (that codes for Leu) in eight exons of position of 229 in the FRMD7 gene, c.686G>T. Two other reports of missense mutation of the position 229 have been described in FRMD7 (17, 18). One of them is a missense c.686C>G mutation in exon 8 of the FRMD7 gene, which results in the substitution of Gly(G) for Arg(R) at amino acid position 229 (p.R229G) (17). The other is a missense c.685C>T mutation, which causes the substitution of Arg (R) with Cys (C) at position 229 (p.R229C) in exon 8 of the FRMD7 gene (18). The missense mutations in position 229 of FRMD7 are mostly deleterious and attributed to the pathogenicity (18).The FRMD7 gene, located in chromosome Xq26.2, comprises 12 exons and encodes a polypeptide containing 714 residues (19). The FRMD7 protein, containing FERM-N, FERM-M, FERM-C, and FA structural domains, is a member of the 4.1 superfamily (19, 20). The identified novel mutation was located in the FERM-C domain, which has been reported to have the highest number of mutations compared with all other domains (20). This mutation was also detected in her mother, maternal grandmother, and two sisters of her maternal grandmother.

In our report family, nystagmus was inherited as an apparent X-linked dominant disorder with incomplete penetrance. Among the tested family members, there were four affected females and two obligate female carriers. Studies have been reported X-linked congenital idiopathic nystagmus pedigrees could be represented a penetrance in the range of 30–100% in female members (21, 22). The possible mechanisms for this variable penetrance include skewed X inactivation, genetic modifiers, regulation of other genes, and other factors that influence ocular motor development (22–26).

FRMD7 is mainly detected in neuronal tissue within the afferent arms of the vestibulo-ocular reflex consisting of the optic vesicle, cranial nerve VIII, vestibular ganglia, developing neural retina, and ventricular zone of the optic stalk. It is also selectively expressed in starburst amacrine cells (7, 27, 28). It is involved in the regulation of eye movement, neuronal morphogenesis, synapse function, and neurite growth (29). The FERM domain and the FERM-adjacent domain control plasma membrane and actin cytoskeleton organization suggesting that they are essential to the development of neural system and the brain region that control eye movement (30).

Several FRMD7 isoforms have been shown to play important roles during neuronal differentiation and development. The original form of FRMD7 (FRMD7-FL) and its splice variants FRMD7-S and FRMD7_SV2 are involved in the neuronal development process (29) (31). A search performed on the BLAST tool showed that the FRMD7 protein shared a close homology with FARP1 and FARP2, which modulate the length and the degree of branching of neurites in rat embryonic cortical neurons and reorganize the cytoskeleton (32, 33).

Evidence shows that FRMD7 mutations are associated with the development of nystagmus (34). Besides, FRMD7 mutations could alter retinal direction selectivity, diminish responsiveness at higher stimulus speeds, and eliminate overrepresentation of posterior-motion-preferring cortical cells (35), thereby cause retinal neuron migratory defects such as foveal hypoplasia (36). In addition, they could affect starburst amacrine cells and impair the development of visual motion responses in superior colliculus neurons downstream of the retina (37). This disrupts axogenesis, dendritogenesis, and neuronal guidance in brain areas involved in the control of eye movement (30). FRMD7 promoted neurite elongation by modulating actin cytoskeleton whereas FRMD7 gene knockdown led to a significant reduction in overall neurite length (34, 38), further contributing to the development of neuronal circuit asymmetry and neurological disorders (28, 39). Mutations associated with ICN include missense mutations, null mutations, deletions and insertions, and frameshift mutations. A series of FRMD7 gene mutations linked to ICN are summarized in **Table 1**.

**Table 1:** Summary of the cases of FRMD7 mutation in ICN.

Year	Author	location	Origin	Mutation type	Nucleotide change	Protein change	References
2020	Fengqi Wang	Exon 12	China	frameshift variant	c.1419_1422dup	p.Tyr475fs	(40)
2019	Naihong Yan	Exon 7	China	splice	c.498-3C > T		(41)
2019	Zhe Wang	Exon 9	China	missense	c.805 A > C	p.Lys269Gln, K269Q	(42)
2018	Yanghui Xiu	Exon 9	China	missense	c.886G>T	p.G296C	(43)
2017	Dayong Bai	Exon 11	China	frameshift	c.999delT	p.H333fs	(18)
2017	Dayong Bai	Exon 4	China	missense	c.284G>T	p.R95M	(18)
2017	Dayong Bai	Exon 4	China	shear	c.206-1G>A	splicing	(18)
2017	Dayong Bai	Exon 3	China	shear	c.162+2T>C	splicing	(18)
2017	Dayong Bai	Exon 9	China	missense	c.782G>A	p.R261Q	(18)
2017	Dayong Bai	Exon 9	China	missense	c.811T>C	p.C271R	(18)
2017	Dayong Bai	Exon 7	China	missense	c.586G>A	p.D196N	(18)
2017	Dayong Bai	Exon 10	China	missense	c.A973G	p.R325G	(18)
2017	Dayong Bai	Exon 7	China	missense	c.521A>T	p.D174V	(18)
2017	Dayong Bai	Exon 9	China	missense	c.T766A	p.F256I	(18)
2017	Dayong Bai	Exon 8	China	missense	c.685C>T	p.R229C	(18)
2017	Dayong Bai	Exon 1	China	non frameshift	c.41_43delAGA	p.Lys14del	(18)
2017	Xiuhua Jia		China		c.41_43delAGA	p.13−15delK	(44)
2017	Xiuhua Jia		China		c.473T>A	p.I158N	(44)
2017	Xiuhua Jia		China		c.605T>A	p.I202N	(44)
2017	Xiuhua Jia		China		c.580G>T	p.A194S	(44)
2017	Xiuhua Jia		China		c.811T>A	p.C271S	(44)
2017	Xiuhua Jia		China		c.1493insA	p.Y498X	(44)
2017	Xiuhua Jia		China	slice mutation	c.57+1G>A		(44)
2016	Hui Zhao	Exon12	China	nonsense	c.1090C>T	p.Q364X	(45)
2016	Hui Zhao	Exon10	China	missense	c.781C>G	p.R261G	(45)
2015	Basamat AlMoallem	Exon 12	Belgium	frameshift	c.2036del	p.(Leu679Argfs*8)	(46)
2015	Basamat AlMoallem	Exon 9	Belgium	missense	c.801C>A	p.(Phe267Leu)	(46)
2015	Basamat AlMoallem		Belgium	splice-site	c.497+5G>A		(46)
2015	Basamat AlMoallem	Exon 2	Belgium	missense	c.70G>A	p.(Gly24Arg)	(46)
2015	Basamat AlMoallem	Exon 9	Belgium	missense	c.886G>C	p.(Gly296Arg)	(46)
2015	Basamat AlMoallem	Exon 10	Belgium	nonsense	c.910C>T	p.(Arg304*)	(46)
2015	Basamat AlMoallem	Exon 8	Belgium	frameshift	c.660del	p.(Asn221Ilefs*11)	(46)
2015	Basamat AlMoallem	Exon 9	Belgium	missense variant	c.875T>C	p.L292P	(46)
2015	Shashank Gupta	Exon 7	North Indian	missense	c.556A>G	p.M186V	(47)
2015	Tomohiro Kohmoto	Exon 9	Japan	missense variant	c.875T>C	p.L292P	(48)
2015	Jae-Hwan Choi		Korean	frameshift	c.1A>G	p.Met1Val	(49)
2014	Xiao Zhang	Exon 9	China	missense	c.780C > A	p.S260R	(50)
2014	Xiao Zhang	Exon 12	China	nonsense	c.1458 C > T	p.Q487X	(50)
2014	Xiao Zhang	Exon 12	China	deletion	c.1645del G	p.V549YfsX554	(50)
2014	Zhe Wan Et.al	Exon 9	China	missense	c.805 A > C	p.Lys269Gln, K269Q	(42)
2013	Yihua Zhu	Exon 9	China		c.719T > C	p.I240T	(51)
2013	Zhirong Liu	Exon 6	China	missense	c.635T>C		(52)
2013	Feng-wei Song	Exon11	China	frameshift	c.980_983delATTA	p.H327PfsX353	(53)
2013	Feng-wei Song	Exon11	China	frameshift	c.986C>A	p.P329E	(53)
2012	Uppala Radhakrishna	Exon 10	Switzerland	missense	c.A917G	Q305R	(54)
2011	Ningdong Li	Exon7	China		c. 623A>G	p. H208R	(55)
2011	Mervyn G. Thomas		Ireland&Gernamy	missense	c.691T>G	p.L231V	(27)
2011	Mervyn G. Thomas		Ireland	missense	c.70G>A	p.G24R	(27)
2011	Mervyn G. Thomas		England	missense	c.812G>A	p.C271Y	(27)
2011	Mervyn G. Thomas		England	missense	c.796G>C	p.A266P	(27)
2011	Mervyn G. Thomas		England	nonsense	c.1003C>T	p.R335*	(27)
2011	Mervyn G. Thomas		India	nonsense	c.1003C>T	p.R335*	(27)
2011	Mervyn G. Thomas		England	missense	c.676G>A	p.A226T	(27)
2011	Mervyn G. Thomas		England	missense	c.47T>C	p.F16S	(27)
2011	Mervyn G. Thomas		England	splice	c.58-1G>A		(27)
2011	Mervyn G. Thomas		Romania	missense	c.1019C>T	p.S340L	(27)
2011	Mervyn G. Thomas		England	missense	c.811T>A	p.C271S	(27)
2011	Wei Du	Exon 12	China	frameshift	c.1486–1489delTTTT	p.F497fs26X	(56)
2008	Xiang He	Exon 9	China		c.812G>T	p.C271F	(57)
2008	Ningdong Li	Exon2	China	missense	70 G>T	p.G24W	(58)
2008	Ningdong Li	Exon8	China		c.689–690delAG	p.Ser232del	(58)
2008	Ningdong Li	Exon9	China	missense	c. 782G>A	p.R260Q	(58)
2008	Ningdong Li	Exon9	China	missense	c. 812G>T	p. C271F	(58)
2008	Ningdong Li	Exon10	China	nonsense	c. 910C>T	R303X	(58)
2008	Xiang He	Exon 12	China	frameshift	c.1274- 1275delTG	p.428X	(59)
2008	Yuksel Kaplan	Exon 8	Turkey	missense	c.686C>G	p.R229G	(17)
2007	Qingjiong Zhang	Exon1	China		c.41-43delAGA	p.Lys14de	(60)
2007	Qingjiong Zhang	Exon2	China		c.70G>A	p.Gly24Arg	(60)
2007	Qingjiong Zhang	Exon6	China		c.436C>T	p.Arg146Trp	(60)
2007	Qingjiong Zhang	Exon8	China		c.685C>T	p.Arg229Cys	(60)
2007	Daniel F Schorderet	Exon 2	Switzerland	nonsense	c.58C>T	p.Q20X	(61)
2007	Daniel F Schorderet	Exon 9	Switzerland	missense	c.824A>C	p.H275P	(61)
2007	Daniel F Schorderet	intron 1	Switzerland	splice	c.57+5G>A		(61)
2007	Daniel F Schorderet	intron 6	Switzerland	splice	c.676-2A>G		(61)
2007	Daniel F Schorderet	Intron 3	Switzerland	variant	c.206-20T>C		(61)
2007	Daniel F Schorderet	Intron 5	Switzerland	variant	c.383-11G>A		(61)
2007	Daniel F Schorderet	Exon 8	Switzerland	missense	c.673T>G	p.W225G	(61)
2007	Alan Shiels	Exon6	USA	missense	c.425T>G	p.L142R	(62)
2007	Baorong Zhang		China	missense	c.781C>G	p.R261G	(63)
2006	Patrick S Tarpey		Ireland	missense	G70A, G24R		(7)
2006	Patrick S Tarpey		England	truncating	IVS2+5G>A		(7)
2006	Patrick S Tarpey		England	truncating	IVS3+2 T>G		(7)
2006	Patrick S Tarpey	Exon4	England	silent	G252A, V84V		(7)
2006	Patrick S Tarpey		England, England	truncating	IVS4+1G>A		(7)
2006	Patrick S Tarpey		Ireland	missense	T425G, L142R		(7)
2006	Patrick S Tarpey		Madagascar	truncating	IVS7+1G>C		(7)
2006	Patrick S Tarpey		Italy-Germany	truncating	C601T, Q201X		(7)
2006	Patrick S Tarpey		Ireland -Germany	missense	T691G, L231V		(7)
2006	Patrick S Tarpey		England, England	missense	G796C, A266P		(7)
2006	Patrick S Tarpey		Scotland	missense	G812A, C271Y		(7)
2006	Patrick S Tarpey		Austria	truncating	887delG, G296fs		(7)
2006	Patrick S Tarpey		England	missense	A902G, Y301C		(7)
2006	Patrick S Tarpey		England, India, England	truncating	C1003T, R335X		(7)
2006	Patrick S Tarpey		Germany	truncating	IVS11+1G>C		(7)
2006	Patrick S Tarpey		England	deletion	41_43delAGA, 14delI		(7)
2006	Patrick S Tarpey		Austria	missense	G71A, G24E		(7)
2006	Patrick S Tarpey		England	truncating	479insT, 160fs		(7)
2006	Patrick S Tarpey		England	missense	A661G, N221D		(7)
2006	Patrick S Tarpey		England	missense	G676A, A226T		(7)
2006	Patrick S Tarpey		Romania	missense	C1019T, S340L		(7)
2006	Patrick S Tarpey		England	truncating	1262delC, 421fs		(7)

In conclusion, our study adds to the spectrum of FRMD7 mutations associated with X-linked ICN. We show that FRMD7 mutations underlie the development of X-linked ICN. Surgery could be an effective treatment approach to correct persistent anomalous head posture in ICN patients. However, the molecular mechanisms by which genetic variations cause ICN are unknown, and therefore, further investigations are required to reveal detailed mechanisms that drive the pathogenesis of this hereditary ocular disease.

This study has several potential limitations. First, only one Chinese family was investigated in the study. In addition, we did not examine protein and cell levels to clarify the pathological mechanism by which FRMD7 mutation led to ICN. Furthermore, the follow-up duration was short. Therefore, further studies with longer follow-up duration are needed to validate the efficacy of surgical therapy in ICN patients.

## Data availability statement

The original contributions presented in the study are included in the article/supplementary materials. Further inquiries can be directed to the corresponding author.

## Ethics statement

The studies involving human participants were reviewed and approved by West China Hospital of Sichuan University. Written informed consent to participate in this study was provided by the participants’ legal guardian/next of kin. Written informed consent was obtained from the individual(s), and minor(s)’ legal guardian/next of kin, for the publication of any potentially identifiable images or data included in this article.

## Author contributions

XJ and FL carried out the experiments, prepared the figures, and drafted the manuscript. MW performed the gene test. ML and LL participated in its design and edited the manuscript. All authors contributed to the article and approved the submitted version.
